# Supplementary material for: U7 small nuclear RNA splice-switching therapeutics for STMN2 and UNC13A in Amyotrophic Lateral Sclerosis
Source: bioRxiv. 2025 Nov 30:2025.11.26.690143. Preprint. [Version 1] doi: 10.1101/2025.11.26.690143 (PMC12697360; doi:10.1101/2025.11.26.690143)
Supplement: Supplement 1 [file media-1.pdf]

Supplementary Information

U7 small nuclear RNA splice-switching therapeutics for STMN2 and UNC13A in Amyotrophic Lateral Sclerosis

Puja R. Mehta[1,8], Tomas Solomon[2,8], Sarah Pickles[3,8], Peter Harley[1], Michela Barioglio[1], Christoph Schweingruber[2], Alessandro Marrero-Gagliardi[1], Yujing Gao[2], Francesca Mattedi[1], Simone Barattucci[1], Lilian Tsai-Wei Lin[3], Eugeni Ryadnov[1], Matteo Zanovello[1], Alexander J. Cammack[4,5], Adrian M. Isaacs[4,5], Juan Burrone[6], Christopher E. Shaw[2], Matthew J. Keuss[1], Leonard Petrucelli[3,9], Pietro Fratta[1,7,9] & Marc-David Ruepp[2,9]

[1]Department of Neuromuscular Diseases, UCL Queen Square Institute of Neurology, UCL Queen Square Motor Neuron Disease Centre, London, UK
[2]UK Dementia Research Institute, Institute of Psychiatry, Psychology & Neuroscience, King's College London, London, UK
[3]Department of Neuroscience, Mayo Clinic, Jacksonville, FL, USA
[4]Dept of Neurodegenerative Disease, UCL Queen Square Institute of Neurology, London, UK
[5]UK Dementia Research Institute at UCL, London, UK
[6]Centre for Developmental Neurobiology and MRC Centre for Neurodevelopmental Disorders, King's College London, London, UK
[7]Francis Crick Institute, London, UK
[8]These authors contributed equally
[9]Correspondence should be addressed to petrucelli.leonard@mayo.edu, p.fratta@ucl.ac.uk or marc-david.ruepp@kcl.ac.uk

Supplementary Information Guide

**Supplementary figure 1:**	tU7 screening information
**Supplementary figure 2:**	Additional quantification of transcript and protein changes following tU7 Treatment in neurons and humanized Mice
**Supplementary table 1:**	U7 SmOPT snRNA sequences
**Supplementary table 2:**	Primer sequences/qPCR assay information

Supplementary Figure S1

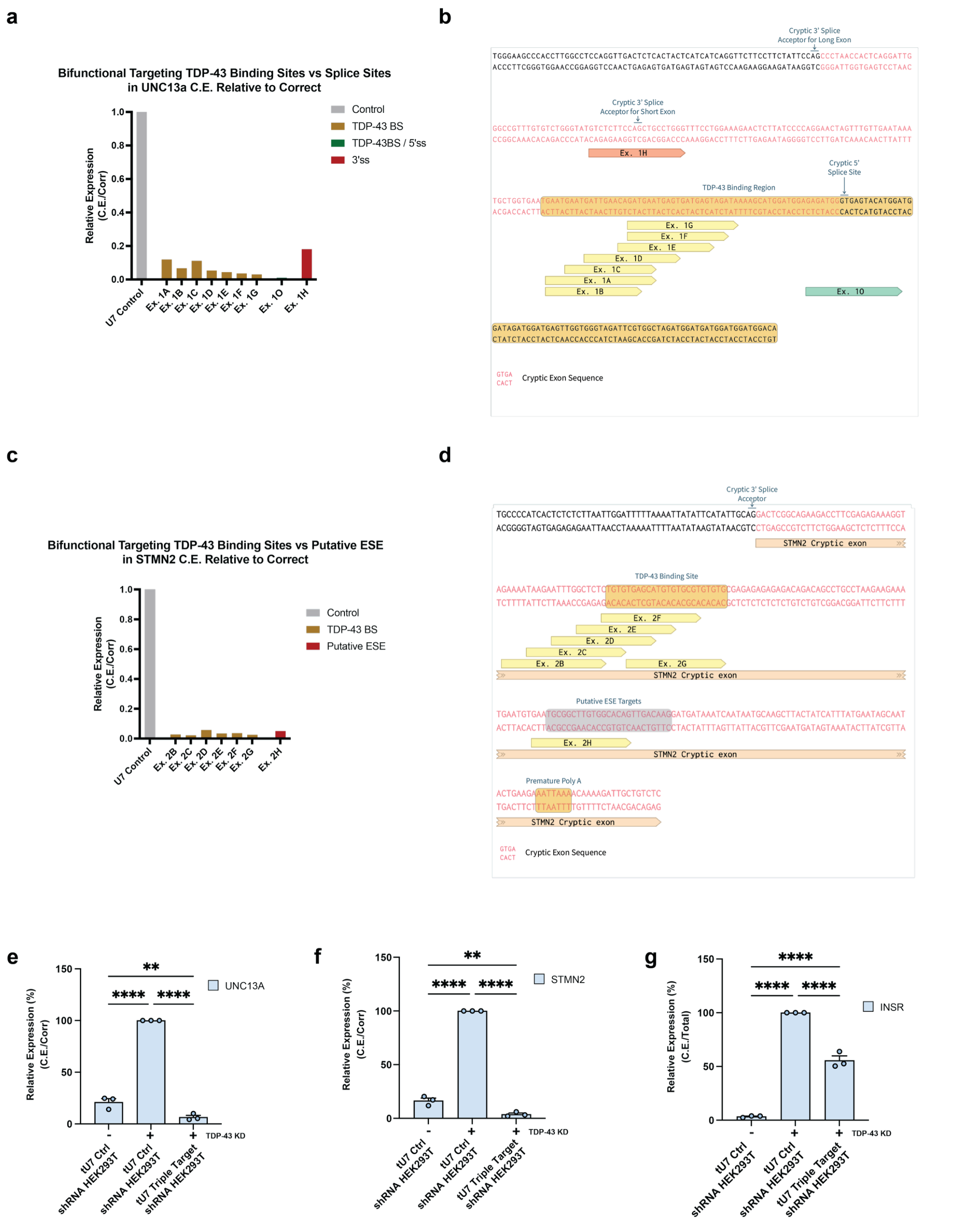

Supplementary Figure 1: a, c Ratio of cryptic exon splicing to correct splicing of the UNC13A (**a**) and STMN2 (**c**) minigene in the presence of different modified U7 snRNA constructs containing either an antisense sequence that targets the 5'-splice site, TDP-43 binding region, 3'-splice site or putative exonic splicing enhancer (ESE) tested in TDP-depleted 293T cells with UNC13A or STMN2 minigene. n = 1. **b, d** Shows the TDP-43 regulated UNC13A (**b**) and STMN2 (**d**) cryptic exon target and flanking regions annotated with splicing elements and tU7 antisense binding regions for screened constructs. **e, f, g** Ratio of cryptic to correctly or total (mean ± SEM)

transcript RT-qPCR levels of *UNC13A* (**e**), *STMN2* (**f**) and *INSR* (**g**) mature mRNA in the TDP-depleted 293T cells transfected with STMN2 and UNC13A minigenes upon transfection with non-targeting control (tU7 Ctrl) or a combined vector comprising multiple U7 constructs pMA-3x-U7SmOPT (tU7 Triple Target). The 3x-tU7SmOPT construct contains three U7 constructs (Ex.1O, Ex. 2C, tU7 INSR, see Table S1) in tandem each comprising a different antisense sequence complementary to UNC13A, STMN2 and INSR cryptic exons. Results are normalised to tU7 Ctrl without TDP-43 KD. n=3. Statistical significance was determined by one-way ANOVAs with Tukey multiple comparison (*$p < 0.05$; **$p < 0.01$; ***$p < 0.001$; ****$p < 0.0001$).

Supplementary Figure S2

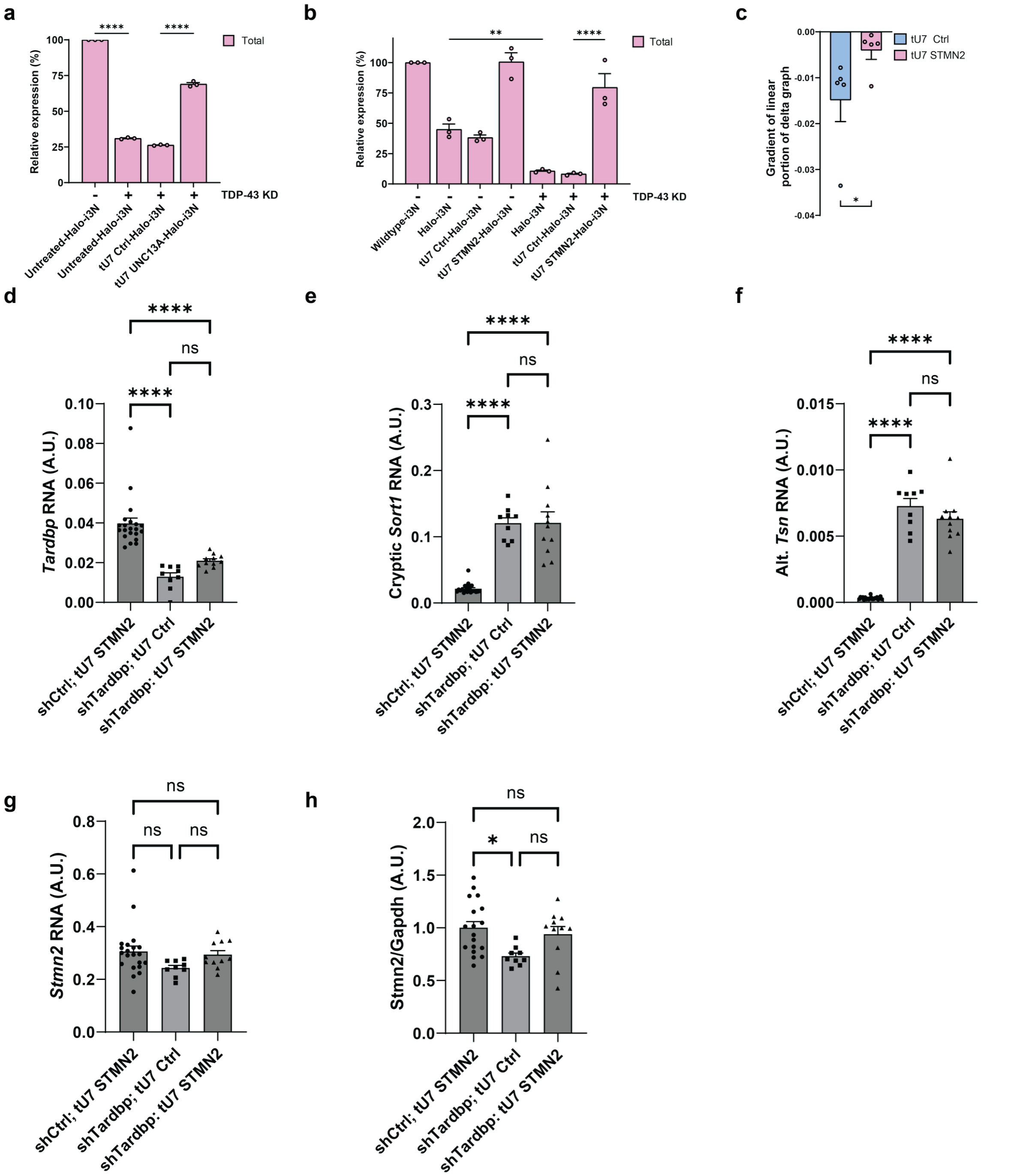

Supplementary Figure 2: a, Quantification (mean ± SEM) of RT-qPCR levels of total *UNC13A* **(a)** and *STMN2* **(b)** transcripts shows successful rescue after respective tU7 treatment. *n* = 3. **c,** Quantification (mean ± SEM) of the gradient of the delta curves' linear portion in **Fig. 2n** for tU7 Ctrl and tU7 STMN2-expressing i3Neurons, showing that the tU7 STMN2, rescued TDP-43 loss-dependent neurite outgrowth impairment. *n* = 5. **d-h,** RNA measured form the forebrains of 1.5-month-old humanized $Stmn2^{+/+}$ mice ICV-injected with AAV expressing shRNA and tU7 snRNA, *shCtrl;* tU7 STMN2 (*n* = 18-21), *shTardbp;* tU7 Ctrl (*n* = 9), and *shTardbp;* tU7 STMN2 (*n* = 11). d, *Tardbp* RNA, normalised to endogenous controls *Rplp0* and *Gapdh*, as measured by RT-qPCR, was significantly reduced in *shTardbp* compared to s*hCtrl*-treated mice. **e-f,** Cryptic *Sort1* **(e)**

and alternatively spliced Tsn RNA **(f)**, normalised to endogenous controls *Rplp0* and *Gapdh*, as measured byRT-qPCR, were significantly increased in *shTardbp* compared to s*hCtrl*-treated mice. **g,** *Stmn2* RNA, normalised to endogenous controls *Rplp0* and *Gapdh*, as measured by RT-qPCR, was not significantly different in any groups. **h,** Stmn2 protein normalised to Gapdh levels are significantly reduced in *shTardbp*; tU7 Ctrl conditions, but similar in *shTardbp*; tU7 STMN2 compared to *shCtrl*; tU7 STMN2-treated mice. Statistical significance for **a,b, and d-h** was determined by one-way ANOVA followed by Tukey's multiple comparisons test, and for **c** was determined by performing a one-tailed two-sample Wilcoxon signed rank test (*$p < 0.05$; **$p < 0.01$; ***$p < 0.001$; ****$p < 0.0001$; ns = non-significant).

Table S1: U7 SmOPT snRNA sequences

Name	Target Antisense Sequence (Sequences taken forward in bold)	tU7 smOPT snRNA Sequence (hnRNPA1 tail underlined)
tU7 Ctrl	GTGTTACAGCTCTTTTAG	AAUAUGAUAGGGACUUAGGGUGGUGUUACAGCUCUUUUAGAAUUUUUGGAGCAGGUUUUCUGACUUCGGUCGGAAAACCCCU
Ex. 1A	TTCATCTGTTCAATCATTCATTC	AAUAUGAUAGGGACUUAGGGUGUUCAUCUGUUCAAUCAUUCAUUCAAUUUUUGGAGCAGGUUUUCUGACUUCGGUCGGAAAACCCCU
Ex. 1B	ATCTGTTCAATCATTCATTC	AAUAUGAUAGGGACUUAGGGUGAUCUGUUCAAUCAUUCAUUCAAUUUUUGGAGCAGGUUUUCUGACUUCGGUCGGAAAACCCCU
Ex. 1C	TTCATCTGTTCAATCATTC	AAUAUGAUAGGGACUUAGGGUGUUCAUCUGUUCAAUCAUUCAAAUUUUUGGAGCAGGUUUUCUGACUUCGGUCGGAAAACCCCU
Ex. 1D	ACTCATTCATCTGTTCAATC	AAUAUGAUAGGGACUUAGGGUGACUCAUUCAUCUGUUCAAUCAAUUUUUGGAGCAGGUUUUCUGACUUCGGUCGGAAAACCCCU
Ex. 1E	ACTCATCACTCATTCATCTG	AAUAUGAUAGGGACUUAGGGUGACUCAUCACUCAUUCAUCUGAAUUUUUGGAGCAGGUUUUCUGACUUCGGUCGGAAAACCCCU
Ex. 1F	TCTACTCATCACTCATTCATC	AAUAUGAUAGGGACUUAGGGUGUCUACUCAUCACUCAUUCAUCAAUUUUUGGAGCAGGUUUUCUGACUUCGGUCGGAAAACCCCU
Ex. 1G	TATCTACTCATCACTCATTCATC	AAUAUGAUAGGGACUUAGGGUGUAUCUACUCAUCACUCAUUCAUCAAUUUUUGGAGCAGGUUUUCUGACUUCGGUCGGAAAACCCCU
**Ex. 1O**	**TCCATGTACTCACCCATCTC**	**AAUAUGAUAGGGACUUAGGGUGUCCAUGUACUCACCCAUCUCAAUUUUUGGAGCAGGUUUUCUGACUUCGGUCGGAAAACCCCU**
Ex. 1H	CCCAGGCAGCTGGAAGAGAC	AAUAUGAUAGGGACUUAGGGUGCCCAGGCAGCUGGAAGAGACAAUUUUUGGAGCAGGUUUUCUGACUUCGGUCGGAAAACCCCU
Ex. 2B	GAGAGCCAAATTCTTATTTTC	AAUAUGAUAGGGACUUAGGGUGGAGAGCCAAAUUCUUAUUUUCAAUUUUUGGAGCAGGUUUUCUGACUUCGGUCGGAAAACCCCU
**Ex. 2C**	**CACAGAGAGCCAAATTCTTA**	**AAUAUGAUAGGGACUUAGGGUGCACAGAGAGCCAAAUUCUUAAAUUUUUGGAGCAGGUUUUCUGACUUCGGUCGGAAAACCCCU**
Ex. 2D	TGCTCACACAGAGAGCCAAAT	AAUAUGAUAGGGACUUAGGGUGUGCUCACACAGAGAGCCAAAUAAUUUUUGGAGCAGGUUUUCUGACUUCGGUCGGAAAACCCCU
Ex. 2E	CACATGCTCACACAGAGAGC	AAUAUGAUAGGGACUUAGGGUGCACAUGCUCACACAGAGAGCAAUUUUUGGAGCAGGUUUUCUGACUUCGGUCGGAAAACCCCU
Ex. 2F	ACGCACACATGCTCACACAG	AAUAUGAUAGGGACUUAGGGUGACGCACACAUGCUCACACAGAAUUUUUGGAGCAGGUUUUCUGACUUCGGUCGGAAAACCCCU
Ex. 2G	CACACACGCACACATGCTCA	AAUAUGAUAGGGACUUAGGGUGCACACACGCACACAUGCUCAAUUUUUGGAGCAGGUUUUCUGACUUCGGUCGGAAAACCCCU
Ex. 2H	CTGTGCCACAAGCCGCATTC	AAUAUGAUAGGGACUUAGGGUGCUGUGCCACAAGCCGCAUUCAAUUUUUGGAGCAGGUUUUCUGACUUCGGUCGGAAAACCCCU
Ex. 2I	CGAAGGTCTTCTGCCGAGTCCTGC	AAUAUGAUAGGGACUUAGGGUGCGAAGGUCUUCUGCCGAGUCCUGCAAUUUUUGGAGCAGGUUUUCUGACUUCGGUCGGAAAACCCCU
**tU7 INSR**	**CCCGTATCCGGTACTATATG**	**AAUAUGAUAGGGACUUAGGGUGCCCGUAUCCGGUACUAUAUGAAUUUUUGGAGCAGGUUUUCUGACUUCGGUCGGAAAACCCCU**

Table S2: Primer sequence/qPCR assay information

**Primers for validation of HEK 293T 2x shTDP cell line**		
**Target / Section**	**Primer**	**Sequence (5’-3’)**
Sybr *TARDBP*	Forward	AACCGAACAGGACCTGAAAGAG
Sybr *TARDBP*	Reverse	CAGTCACACCATCGTCCATCTATC
Sybr *ACTB*	Forward	TCCATCATGAAGTGTGACGT
Sybr *ACTB*	Reverse	TACTCCTGCTTGCTGATCCAC
**Primers used in HEK 293T 2x shTDP minigene assays**		
**Target / Section**	**Primer**	**Sequence (5’-3’)**
TARDBP	Forward	TCATCCCCAAGCCATTCAGG
TARDBP	Reverse	TGCTTAGGTTCGGCATTGGA
GAPDH	Forward	CCAGAACATCATCCCTGCCT
GAPDH	Reverse	GGTCAGGTCCACCACTGACA
Canonical *UNC13A*	Forward	ACCTGTCTGCATGAGAACCT
Canonical *UNC13A*	Reverse	GGGCTGTCTCATCGTAGTAAAC
Cryptic *UNC13A*	Forward	ATGGATGGAGAGATGGAACCT
Cryptic *UNC13A*	Reverse	GGGCTGTCTCATCGTAGTAAAC
Canonical *STMN2*	Forward	GCTAAAACAGCAATGGCCTAC
Canonical *STMN2*	Reverse	TTGCTTCACTTCCATATCATCG
Cryptic *STMN2*	Forward	GCTAAAACAGCAATGGGACTC
Cryptic *STMN2*	Reverse	GCAGGCTGTCTGTCTCTCTC
Total *INSR*	Forward	TGGGACCGCTTTACGCTTC
Total *INSR*	Reverse	GAGACTGGCTGACTCGTTGAC
Cryptic *INSR*	Forward	CTCTGGGACTGGAGCAAAC
Cryptic *INSR*	Reverse	CATCCCGTATCCGGTAAGG
**Primers used in SH-SY5Y models**		
**Target / Section**	**Primer**	**Sequence (5’-3’)**
UNC13A RT-PCR	Forward	CCGTACCATGTCCAGTACAC
UNC13A RT-PCR	Reverse	AGTAAACCTTCCAGGCATCG
STMN2 RT-PCR	Forward	GCTCTCTCCGCTGCTGTAG
STMN2 RT-PCR	Reverse	CGAGGTTCCGGGTAAAAGCA
STMN2 RT-PCR	Cryptic reverse	CTGTCTCTCTCTCTCGCACA
TARDBP	Forward	TCATCCCCAAGCCATTCAGG
TARDBP	Reverse	TGCTTAGGTTCGGCATTGGA
GAPDH	Forward	CCAGAACATCATCCCTGCCT
GAPDH	Reverse	GGTCAGGTCCACCACTGACA
Correct *UNC13A*	Forward	ACCTGTCTGCATGAGAACCT
Correct *UNC13A*	Reverse	GGGCTGTCTCATCGTAGTAAAC
Cryptic *UNC13A*	Forward	ATGGATGGAGAGATGGAACCT
Cryptic *UNC13A*	Reverse	GGGCTGTCTCATCGTAGTAAAC
Canonical *STMN2*	Forward	GCTAAAACAGCAATGGCCTAC
Canonical *STMN2*	Reverse	TTGCTTCACTTCCATATCATCG
Cryptic *STMN2*	Forward	GCTAAAACAGCAATGGGACTC
Cryptic *STMN2*	Reverse	GCAGGCTGTCTGTCTCTCTC
**Primers for cloning pLVX-EF1a-mCherryT2A-BSD-U7smOPT plasmid**		
**Target / Section**	**Primer**	**Sequence (5’-3’)**
LV inf pLVX Cla	Forward	AGATCCAGTTTATCGATACCAACATAGGAGCTGTGATTGG
LV inf pLVX Cla	Reverse	ATGAATTACTCATCGGCGAGAAAGGAAGGGAAGAAAGC
**Primers/assays used in iPSC-derived models**		
**Target / Section**	**Primer**	**Sequence (5’-3’)**
UNC13A RT-PCR	Forward	GACATCAAATCCCGCGTGAA
UNC13A RT-PCR	Reverse	CATTGATGTTGGCGAGCAGG
STMN2 RT-PCR	Forward	GCTCTCTCCGCTGCTGTAG
STMN2 RT-PCR	Reverse	CGAGGTTCCGGGTAAAAGCA
STMN2 RT-PCR	Cryptic reverse	CTGTCTCTCTCTCTCGCACA

TARDBP RT-qPCR (SYBR Green)	Forward	GATGGTGTGACTGCAAACTTC
TARDBP RT-qPCR (SYBR Green)	Reverse	CAGCTCATCCTCAGTCATGTC
GAPDH RT-qPCR (SYBR Green)	Forward	CACCAGGGCTGCTTTTAACT
GAPDH RT-qPCR (SYBR Green)	Reverse	GACAAGCTTCCCGTTCTCAG
TARDBP RT-qPCR (Taqman; JUN) assay	-	Hs00606522_m1 (Thermo)
GAPDH RT-qPCR (Taqman; JUN) assay	-	GAPDH-Jun 4485713 (Thermo)
STMN2 total RT-qPCR (exon 1-2; Taqman; FAM) assay	-	Hs00199796_m1 (Thermo)
STMN2 correctly spliced RT-qPCR (exon 2-3; Taqman; VIC) assay	-	Hs00975900_m1 (Thermo)
UNC13A total RT-qPCR (exon 25-26; Taqman; FAM) assay	-	Hs00392638_m1 (Thermo)
UNC13A correctly spliced RT-qPCR (exon 20-21; Taqman; VIC) assay	-	Hs00606522_m1 (Thermo)
**Primers used in mouse model experiments**		
**Target / Section**	**Primer**	**Sequence (5’-3’)**
Cryptic *Sort1*	Forward	AAATCCCAGGAGACAAATGC
Cryptic *Sort1*	Reverse	GAGCTGGATTCTGGGACAAG
Alt. *Tsn*	Forward	GGTCTTCCTGGCAGCATTTG
Alt. *Tsn*	Reverse	TTGACAGACAGCCTCGATGC
Tardbp	Forward	GGGGCAATCTGGTATATGTTG
Tardbp	Reverse	TGGACTGCTCTTTTCACTTTCA
Cryptic *Stmn2*	Forward	GCCTTACTCAGACTCCTCTCTC
Cryptic *Stmn2*	Reverse	TCTTCTGCCGAGTCCCATT
Canonical *Stmn2*	Forward	GCAATGGCCTACAAGGAAAA
Canonical *Stmn2*	Reverse	GGTGGCTTCAAGATCAGCTC
Gapdh	Forward	CATGGCCTTCCGTGTTCCTA
Gapdh	Reverse	CCTGCTTCACCACCTTCTTGAT
Rplp0	Forward	ACTGGTCTAGGACCCGAGAAG
Rplp0	Reverse	CTCCCACCTTGTCTCCAGTC
**Primers for genotyping**		
**Target / Section**	**Primer**	**Sequence (5’-3’)**
hStmn2	Forward	TGGATTACAGAATATTTCACTTCCAA
hStmn2 -WT	Reverse	CCCACCCACACACATATTCAC
hStmn2 -Mutant	Reverse	TTGTCAACTGTGCCACAAGC
